# Inference-Based Decisions in a Hidden State Foraging Task: Differential Contributions of Prefrontal Cortical Areas

**DOI:** 10.1016/j.neuron.2020.01.017

**Published:** 2020-04-08

**Authors:** Pietro Vertechi, Eran Lottem, Dario Sarra, Beatriz Godinho, Isaac Treves, Tiago Quendera, Matthijs Nicolai Oude Lohuis, Zachary F. Mainen

**Affiliations:** 1 Champalimaud Research, Champalimaud Centre for the Unknown, 1400-038 Lisbon, Portugal; 2 The Edmond and Lily Safra Center for Brain Sciences, The Hebrew University of Jerusalem, Edmond J. Safra Campus, Givat Ram, 91904 Jerusalem, Israel; 3 Nuffield Department of Clinical Neurosciences, University of Oxford, John Radcliffe Hospital, Oxford OX3 9DU, UK; 4 MIT Department of Brain and Cognitive Sciences, Massachusetts Institute of Technology, 77 Massachusetts Avenue, Room 46-2005, Cambridge, MA 02139-4307, USA; 5 Cognitive and Systems Neuroscience Group, Swammerdam Institute for Life Sciences, Faculty of Science, University of Amsterdam, 1098XH Amsterdam, the Netherlands; 6 Research Priority Area Brain and Cognition, University of Amsterdam, Amsterdam, the Netherlands

**Keywords:** foraging, PFC, inference, state representation, cross-species task

## Abstract

Essential features of the world are often hidden and must be inferred by constructing internal models based on indirect evidence. Here, to study the mechanisms of inference, we establish a foraging task that is naturalistic and easily learned yet can distinguish inference from simpler strategies such as the direct integration of sensory data. We show that both mice and humans learn a strategy consistent with optimal inference of a hidden state. However, humans acquire this strategy more than an order of magnitude faster than mice. Using optogenetics in mice, we show that orbitofrontal and anterior cingulate cortex inactivation impacts task performance, but only orbitofrontal inactivation reverts mice from an inference-based to a stimulus-bound decision strategy. These results establish a cross-species paradigm for studying the problem of inference-based decision making and begins to dissect the network of brain regions crucial for its performance.

## Highlights

• Naturalistic hidden-state foraging task
• Disambiguation between stimulus-bound and inference-based foraging solutions
• Both mice and humans can tune behavior to environment statistics
• Differential role of orbito-frontal cortex and anterior cingulate cortex in inference

## Teaser

Vertechi et al. design a behavioral task to probe inference in mice and humans. They demonstrate that mice and humans adopt inference-based strategies, tuned to the environment statistics. Optogenetic inactivation reveals that the orbito-frontal cortex is necessary for this inference process.

## Introduction

In natural foraging behaviors, animals must continually choose between trying to exploit resources at their current location and leaving to explore another, potentially superior one, at the expense of a possibly costly travel period. Viewed from the perspective of optimal decision-making, the crucial question is when is it best to leave the current site for another one? According to the marginal value theorem, in order to maximize returns, an optimal forager ought to leave its current site when the immediate rate of reward drops below the average rate (Charnov, 1976). However, this elegant solution to the foraging problem only applies in deterministic environments (Kolling et al., 2014), in which both immediate and average reward rates are knowable to the agent. In a more realistic scenario—for example, where rewards are encountered probabilistically—the immediate reward rate is ill-defined and the marginal value theorem does not apply.

One widely used and powerful approach to model decision making in dynamic, stochastic environments is reinforcement learning (RL) (Sutton and Barto, 2018). In RL, the values of different actions (such as leaving a foraging site or staying on) are continuously updated through trial and error, based on their outcomes, allowing agents to adaptively modify their preferences as conditions change. In its simplest form, model-free RL assigns each action with a value that is updated based on its immediate outcome, with no regard to the causal, and often hidden, structure that links actions to outcomes. Although computationally efficient and consistent with a large body of experimental data on both Pavlovian and operant tasks (Eshel et al., 2016, Schultz et al., 1997), model-free RL is not the best available strategy in many situations. Consider, for instance, a lion that has just successfully captured prey. If the fact that in doing this it has most likely scared away all other animals is ignored, the lion may continue to hunt in the same region, wasting a considerable amount of time searching for the now long-gone prey. Conversely, things may turn out badly for a zebra if it assumes that its current foraging ground was safe (that is, lion-free) just because it had not seen a lion yet in its immediate surroundings. What these examples illustrate is that relying solely on recent outcomes, while ignoring causal structures in the world, may have suboptimal (if not catastrophic) consequences. Instead, in structure learning (Boyen et al., 1999, Braun et al., 2010, Pearl, 1991), a form of inference-based RL, agents choose actions based on their beliefs about the current state of the world, which is determined by both incoming sensory evidence (such as outcomes) and knowledge of the underlying causal structure of the environment. How humans and animals implement such strategies remains an important and poorly understood question (Daw et al., 2011, Niv et al., 2015, Starkweather et al., 2018). The study of the neural mechanisms underlying flexible, integrative behavior has drawn special attention to the prefrontal cortex and the computational role of its different areas. Although the mapping of the rodent prefrontal cortex has not reached a consensus, here, we adopt the description of Uylings and van Eden (1990), which defines as rat prefrontal cortex those areas comparable to the primate prefrontal cortex in terms of thalamic reciprocal connections, corticocortical connections, and functional aspects, including the orbitofrontal cortex (OFC) and the anterior cingulate cortex (ACC). It has been suggested that the OFC is crucial for hidden state representation, and hence for inference-based decisions. For example, in both rats and primates, lesions or inhibition of OFC impairs subjects’ ability to adjust their behavior in reversal learning tasks, where the depletion of a previously rewarding site (or the futility of a previously rewarding action) may be viewed as a change in the (hidden) state of the world (Wilson et al., 2014), even though this result may be technique-dependent: Rudebeck et al. (2013) found that aspiration lesions (that also damage passing fibers), but not excitotoxic lesions, of monkey OFC impaired reversal learning. The adjacent ACC, often considered part of the rodent medial prefrontal cortex (Starkweather et al., 2018, Tervo et al., 2014), has been implicated in monitoring value during foraging (Hayden et al., 2011, Kolling et al., 2012) and could be responsible for encoding the value of alternative options (Kolling et al., 2016) and changing behavior based on the decreasing value of the current option (Shima and Tanji, 1998, Williams et al., 2004).

Here, we describe a foraging task in which subjects may seek rewards at either one of two foraging sites. This task has a special hidden structure: at any given moment, only one of the sites can deliver rewards and the site of the rewards switches with a certain probability after each foraging attempt. Importantly, even when reward is available, it is not delivered for every attempt, but rather with a probability less than 1. This makes the task a partially observable Markov decision process (POMDP): the true state of world (i.e., the identity of the rewarding site) is hidden and subjects must infer it based on noisy observations. A defining feature of this task, due to the hidden structure, is the asymmetry of the evidence provided by rewards and failures (unrewarded attempts): a single failure provides partial evidence in favor of a site switch, whereas a single reward provides full certainty that the current site is rewarding. A “stimulus-bound” agent, in the sense of Wilson et al. (2014), would assign value to observable states (being on the left or on the right or the other foraging site) by linearly combining rewards and failures. Such a process does not capture the essential asymmetry of the task. Ten rewards are much better than one reward in terms of value, but under optimal inference, one single reward is as informative as ten, because it already gives absolute certainty that the current site is active. Thus, leaving decisions under stimulus-bound and inference-based strategies in this task will be qualitatively different. A stimulus-bound agent will become more persistent the more rewards it has received at a site, whereas an inference-based agent will not show such an effect. We found that both mice and humans display hallmarks of inference in the performance of a foraging task and are able to build a non-trivial representation of task space. We further show that optogenetic inhibition of the OFC in mice selectively disrupts optimal inference behavior, biasing mice toward a sub-optimal stimulus-bound strategy. Similar inhibition of the adjacent ACC results in delayed leaving decisions but does not disrupt the inference process itself, suggesting a specific role of OFC in this important cognitive function.

## Results

### A Probabilistic Foraging Task Can Dissociate Value or Evidence Accumulation

**Figure 1:**
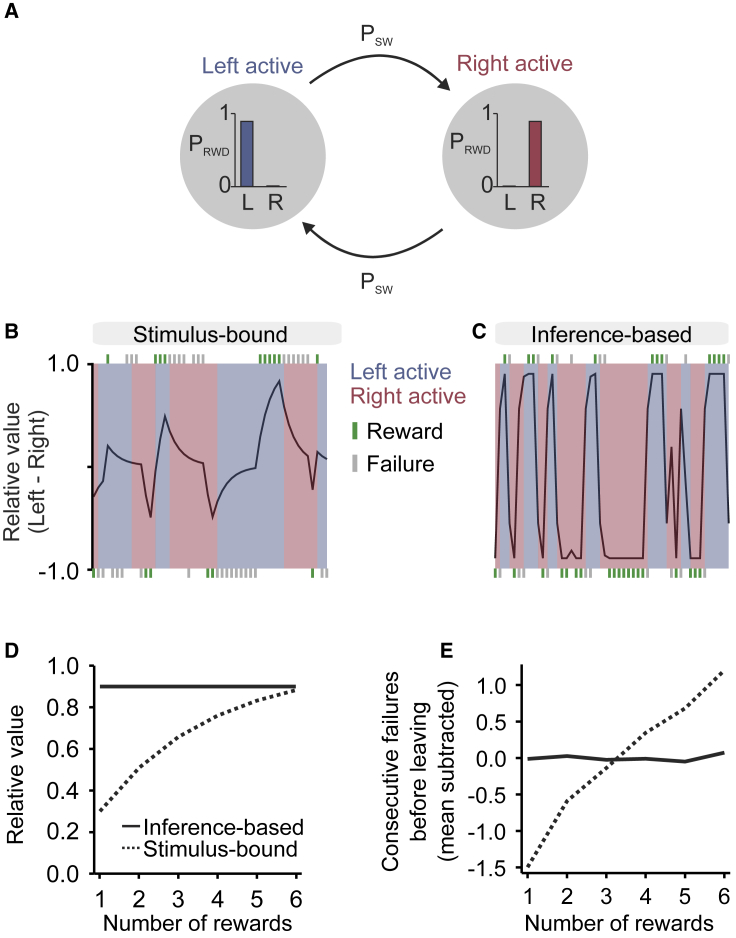
A Probabilistic Foraging Task Can Dissociate Stimulus-Bound from Inference-Based Evidence Accumulation (A) Formally, the task is a hidden Markov model with *LeftActive* and *RightActive* states. It has two parameters: probability of reward given state and probability of state transition. (B and C) Estimated relative value (left minus right) as a function of trial history (rewards in green, failures in gray) in the stimulus-bound model (B) and inference-based model (C), respectively. Shaded patches indicate actual state. (D) Effect of rewards on relative value in stimulus-bound and inference-based models: the two models are simulated in a trial with only rewards on the same site. Relative value increases with reward number in the stimulus-bound but not in the inference-based model. (E) Consecutive failures before leaving (normalized subtractively) as a function of reward number in a simulated data of stimulus-bound and inference-based models: reward number has an effect on consecutive failures in the stimulus-bound but not in the inference-based model.

We developed a self-paced probabilistic foraging task. Subjects sought rewards by actively probing a foraging site. Each try at the active site yielded reward with probability *p*_*RWD*_, and could cause a switch with probability *p*_*SW*_ (Figure 1A). After a state switch, to obtain more rewards, subjects needed to travel to a second site at some distance and therefore bear a travel cost. Subjects were thus tasked with inferring a hidden state of the current site through a sequence of observations of stochastic events (rewards and failures). There are actually many ways of integrating rewards and failures to form a decision. In a stimulus-bound process, the relative value of the left site with respect to the right site *V* = *V*_*LEFT*_ − *V*_*RIGHT*_ would increase gradually with left rewards, decrease gradually with right rewards, and decay to 0 with failures (Figure 1B). In formulas, given a decay coefficient γ, a reward indicator *r*_*t*_, a site indicator *s*_*t*_ (1 for left, −1 for right), and signed outcomes *o*_*t*_ = *r*_*t*_ × *s*_*t*_:

(Equation 1) $$V_{t+1}=\left(1-\gamma \right)V_{t}+\gamma o_{t+1}.$$

On the other hand, an agent that is aware of the structure of the task—the fact that a hidden state determines which site is rewarding at any time—could use rewards and failures differently, allowing it to better infer whether the current foraging site is active or inactive. The relative value would then be:

(Equation 2) $$V_{t}=p_{RWD}\left(P\left(Lef\;tActive|r_{1},s_{1},\;\ldots ,r_{t},s_{t})-P(RightActive|r_{1},s_{1},\ldots ,r_{t},s_{t}\right)\right),$$

(see STAR Methods for a detailed treatment of the probability computation). Unlike the stimulus-bound mechanism in Figure 1B, this process is able to track effectively the rapidly evolving value of the foraging sites (Figure 1C). Both accumulation processes can be used as generative models of the behavior by defining the probability of staying on, e.g., the left site as a sigmoidal function of the relative value:

(Equation 3) P(NextLeft|V,s)=σ(β(V+s·T)),

where β represents a softmax parameter (the higher it is, the more deterministic the behavior), and *T* represents the staying bias (when the value of the left and the right site are estimated as equal, the subject should still prefer to stay to avoid the travel cost).

Both models predict that the probability of leaving increases with the number of consecutive failures. However, the effect of a reward is very different between them. In a stimulus-bound model, the probability of leaving decreases with the number of rewards, as each reward contributes to the accumulated value. In the inference-based model, it does not, because a single reward is sufficient to deduce with certainty that the current site is active (Figure 1D). Thus, a simple test of whether subjects are using inference is to check whether the number of failures before leaving changes with the number of preceding rewards (Figure 1E).

### Mice Accumulate Evidence and Not Rewards

**Figure 2:**
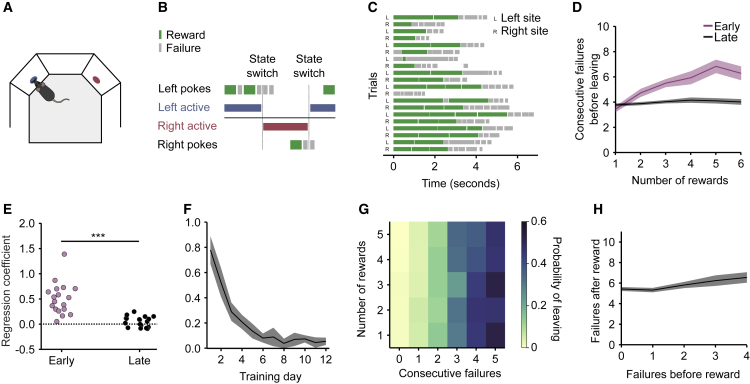
Mice Accumulate Inferred Evidence for State Switches and Not Site Value (A) Schematic of rodent task. Mice shuttle back and forth between two reward sites to obtain water rewards. (B) Example sequence of pokes. Pokes in the correct site can be rewarded or not, whereas pokes in the incorrect site are never rewarded. Following a state switch, the animals need to travel to the other site to obtain more rewards. (C) Example behavior: sequence of poke bouts (i.e., trials) with rewards in green and failures in gray. (D) Consecutive failures before leaving as a function of reward number in early training (days 1 to 3, purple) compared with late training (days 10 to 12, black). Solid line represents mean and sha. (E) Slope coefficient in ConsecutiveFailures∼1+RewardNumber for early training and late training. Slope coefficient is higher in early trials, likelihood ratio test on linear mixed-effect model ConsecutiveFailures∼1+RewardNumber+Early+RewardNumber&Early+(1|MouseID) versus a null model with no interaction: p < 1e−10, n = 18 mice (see STAR Methods for a description of the formula notation). (F) Evolution of reward number coefficient across days. Solid line and shaded area represent mean and across animals. Solid line and shaded area represent mean and SEM across animals. (G) Probability of leaving as a function of number of rewards and consecutive failures in late training. (H) Failures after reward as a function of failure before reward in trials with only one reward in a more difficult protocol. Solid line and shaded area represent mean and across animals. Solid line and shaded area represent mean and SEM across animals. See also Video S1. Video S1. Murine Foraging Task, Related to Figure 2An example mouse performing the hidden-state foraging task inside a behavioral box, viewed from the top

We first developed the hidden state foraging task as a rodent behavioral task (Figure 2A) in which mice had to nose-poke at one of two possible ports to obtain water rewards (2 μL each). We trained 18 C57BL/6 wild-type mice of 2 months age for 12 days in a baseline protocol with *p*_*RWD*_ = 0.9 and *p*_*SW*_ = 0.3 and observed the effect of rewards on behavior during learning.

Because our task was a foraging style task, the mapping to choice and feedback are not present in a trial. Each trial contains a number of pokes, each of which contains feedback in the form of water (or not), as illustrated in Figure 2B. The choice of the mouse is whether to continue to poke (exploit) at the current site or whether to leave (explore), a choice it makes after each poke. The only incorrect choice is to leave to the other site before the state has switched. When the mouse switches site too early, no rewards will be emitted by the other port; the mouse is obliged to return to the original port and continue to poke. Mice made only ∼2.25% ± 0.47% (n = 18 mice) errors on average. An example of the behavior of a trained animal is shown in Figure 2C (see Figure S1 for summary statistics of the durations of the various task epochs).

Mice tended to alternate bouts of pokes at a given site (6.98 ± 0.14 pokes per bout, n = 18 mice) with trips to the opposite site, producing a natural segmentation in trials (i.e., poke bouts on the same site). This presumably reflects the clear asymmetry in time cost between nose-poking again on the same site, a very cheap action (Figure S1, inter-poke interval = 0.16 ± 0.025 s, unrewarded poke duration = 0.33 ± 0.006 s, n = 18 mice), and switching site, a much more expensive option (Figure S1, 3.15 ± 0.18 s, n = 18 mice).

We found that the number of consecutive failures since the last reward (ConsecutiveFailureIndex) was a better predictor of mouse choice than the time spent at the nose poke (TimeSpentAtPort) (Lottem et al., 2018). We fitted two logistic regression models with random effects. Here, and throughout the text, we use Wilkinson notation (Wilkinson and Rogers, 1973) (see STAR Methods for a detailed explanation):

LeavingPort∼1+ConsecutiveFailureIndex+(1|MouseID)

and

LeavingPort∼1+TimeSpentAtPort+(1|MouseID).

The ConsecutiveFailureIndex model was overwhelmingly better (deviance = 10,892) than the TimeSpentAtPort model (deviance = 14,771). As confirmation, we also tested a model that included both predictors:

Leaving∼1+ConsecutiveFailureIndex+TimeSpentAtPort+(1|MouseID),

and only the ConsecutiveFailureIndex had a positive coefficient (0.78 ± 0.013) whereas the TimeSpentAtPort had a small negative coefficient (−0.046 ± 0.006).

In the early part of training, animals were unaware of the structure of the task and exhibited hallmarks of a stimulus-bound strategy: more failures were needed to leave the foraging port in rich foraging bouts, with many rewards before a state switch, compared to poor foraging bouts, with as little as one reward before a state switch. After training, however, the number of rewards had no effect on the number of failures before leaving, consistent with an inference-based strategy (Figure 2D). To quantify this effect at a single animal level, we fitted a linear regression model that predicted the number of consecutive failures before leaving as a function of the number of prior rewards in the current trial (i.e., foraging bout at a given site): ConsecutiveFailures∼1+RewardNumber (Figure 2E). The data show that during the first days of training, there was a strong positive correlation between these two quantities, but with continued training this correlation decayed to zero (Figure 2F). Therefore, experienced mice, unlike naive animals, decide when to leave the foraging site in a manner consistent with inferring a hidden state rather than directly integrating rewards and failures.

As another way of seeing this, a stimulus-bound integration strategy would effectively weigh similarly each reward and failure with opposite signs (see Equation 1). Correct inference instead, given the structure of this task, requires that rewards are weighted nonlinearly (the first counting a lot and subsequent nothing) and differently from failures, which should add linearly. Indeed, in the trained mice, the effect of rewards and failures in shaping the behavior is qualitatively asymmetric in just this way, as can be seen by visualizing the probability of leaving as a function of both reward number and consecutive failures (Figure 2G).

Furthermore, stimulus-bound and inference-based models predict different interactions of rewards with preceding failures. Consider, for example, trials in which the animal receives a single reward: the later the reward, the smaller the value of the current site at the time of reward delivery. In the stimulus-bound model, the received reward value is simply added to the current value estimate, so the later in the train the reward arrives the lower the current value of the port (given that we are assuming only failures before this reward). Therefore, fewer subsequent failures will be tolerated before the animal leaves. On the other hand, in inference-based models a single reward resets the count of accumulated failures up to that point, and therefore the position of the reward (or equivalently the number of failures prior to the reward) has no consequence on subsequent behavior. To test these alternatives, we analyzed how the position of that reward influenced the overall number of failures before leaving in a protocol with lower probability rewards (*p*_*RWD*_ = 0.3 and *p*_*SW*_ = 0.3) and found that the number of failures after the last reward did not decrease when it was preceded by more and more failures, on the contrary it slightly increased (Figure 2H, slope = 0.2 ± 0.06, n = 20 mice), consistent with a resetting effect of reward as predicted by the inference-based model.

### Accumulation of Evidence Is Tuned to Task Parameters

**Figure 3:**
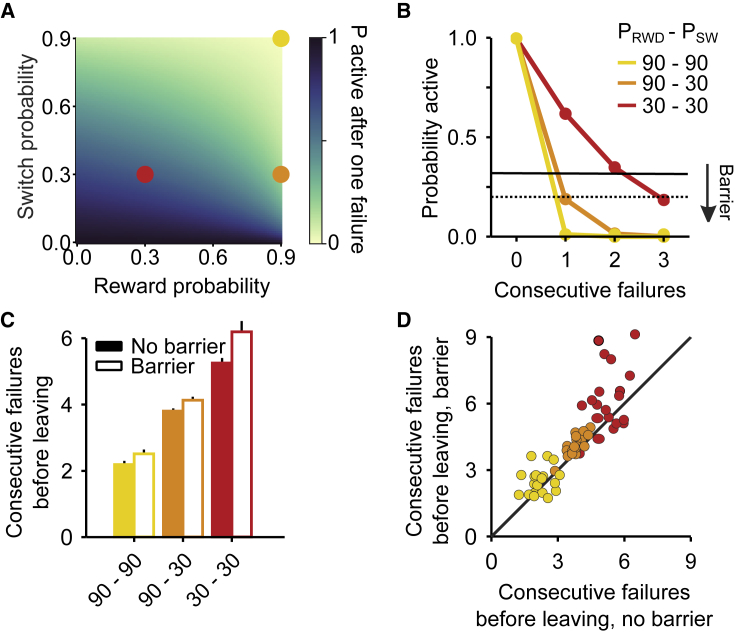
Accumulation of Inferred Evidence Is Tuned to Task Parameters (A) Probability of being on the correct site after a failure as a function of reward probability and transition probability. (B) Probability of being on the correct site as a function of trial history for three protocols (Easy environment: *p*_*RWD*_ = 0.9 and *p*_*SW*_ = 0.9; Medium environment: *p*_*RWD*_ = 0.9 and *p*_*SW*_ = 0.3; Hard environment: *p*_*RWD*_ = 0.3 and *p*_*SW*_ = 0.3). Leaving decisions can be modeled by setting a threshold on this probability that changes as a function of the travel cost (black lines). (C) Consecutive failures before leaving as a function of the environment statistics and barrier condition. Error bars represent SEM across animals. (D) Consecutive failures before leaving split by subject and environment statistics, barrier versus no barrier.

Having found that the foraging behavior of mice is consistent with the accumulation of evidence to infer a hidden world state, we asked whether this inference process is appropriately tuned to the statistics of the foraging environment, represented here by two parameters: reward probability *p*_*RWD*_ and state switch probability *p*_*SW*_. Intuitively, if *p*_*RWD*_ is high, then a single failure is strong evidence in favor of a state switch, leading to a faster accumulation process. Similarly, if *p*_*SW*_ is high, then a failure also carries more evidence in favor of a state switch compared to if it is low (Figure 3A; see STAR Methods for a formal justification of this intuitive argument).

To test this, we trained a separate batch of mice on a set of three different foraging site statistics (Easy environment: *p*_*RWD*_ = 0.9 and *p*_*SW*_ = 0.9; Medium environment: *p*_*RWD*_ = 0.9 and *p*_*SW*_ = 0.3; Hard environment: *p*_*RWD*_ = 0.3 and *p*_*SW*_ = 0.3; see Figure 3B). Because changing the foraging environment’s statistics can affect average reward rates (i.e., average number of rewards per trial), we adjusted the magnitude of individual rewards in order to equalize the amount of reward at a given site before state switch across conditions. As predicted normatively, mice increased the number of failed attempts they would tolerate as the state switching probability and the reward probability dropped (Figures 3C and 3D; difference in failed attempts after last reward in Easy-Medium = −1.61 ± 0.03, difference Hard-Medium = 1.77 ± 0.04, n = 20 mice, likelihood ratio test on ConsecutiveFailures∼1+Protocol+(1|MouseID)versus ConsecutiveFailures∼1+(1|MouseID): p < 1e−10).

An important additional prediction of optimal decision theory in the context of a foraging task is that travel cost should modulate the threshold to leave a given foraging site. To test this, we increased the travel cost by placing a physical barrier between the two locations (travel time without barrier = 1.86 ± 0.13 s, n = 20 mice; travel time with barrier = 2.69 ± 0.13 s). Once again, the accumulation process was modulated consistently with the normative prediction, longer travel times resulting in a longer accumulation process and delayed leaving (Figures 3C and 3D, effect of barrier in number of failed attempts after last reward = 0.42 ± 0.03, n = 20 mice, likelihood ratio test on ConsecutiveFailures∼1+Protocol+Barrier+(1|MouseID) versus ConsecutiveFailures∼1+Protocol+(1|MouseID): p < 1e−10).

### Humans Perform Inference and Tune Behavior to Task Parameters

To test whether our findings were valid across species, we developed a translation of our behavioral assay for human subjects, in the form of a video game, where players would drag a character from one side of a touch screen to the other and tap to achieve points. The statistics of the video game (*p*_*RWD*_ and *p*_*SW*_) were the same as those used in the rodent task.

**Figure 4:**
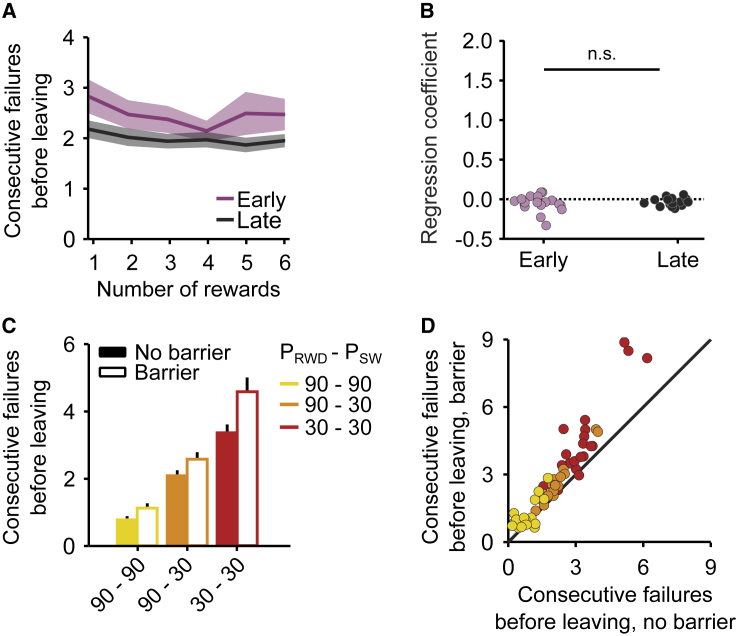
Humans Perform Optimal Inference and Tune Behavior to Task Parameters (A) The number of rewards has little effect on the probability of leaving during both early (purple) and late (black) training. Solid line and shaded area represent mean and across animals. Solid line and shaded area represent mean and SEM across animals. (B) Number of consecutive failures as a function of reward number for human in early versus late part of training. Unlike mice, humans learn the statistics of the environment extremely quickly: slope coefficient is similar (and around 0) in both early and late trials: likelihood ratio test on linear mixed-effect model ConsecutiveFailures~1+RewardNumber+Early+RewardNumber&Early+(1|SubjectID) versus a null model with no interaction: p = 0.45, n = 20 subjects. (C) Consecutive failures before leaving as a function of the environment statistics and barrier condition. Error bars represent SEM across subjects. (D) Consecutive failures before leaving split by subject and environment statistics, barrier versus no barrier. See also Video S2. Video S2. Human Foraging Task, Related to Figure 2A human participant showcasing correct and incorrect transitions, touch screen use, as well as easy, medium, and hard protocols in the hidden-state foraging task.

In humans, we again observed hallmarks of inference-based foraging: the number of rewards had little to no effect on the behavior (Figures 4A and 4B), similar to the behavior of the trained mice. Unlike mice, however, humans needed almost no training to learn this strategy, displaying it from the first session.

Analogously to their rodent counterparts, human subjects modulated their behavior according to reward statistics as well as travel time (here affected by a manipulation in the character’s velocity) consistent with the normative predictions (Figures 4C and 4D, difference in failed attempts after last reward in Easy-Medium = −1.39 ± 0.03, difference Hard-Medium = 1.48 ± 0.03, n = 20 subjects, likelihood ratio test on ConsecutiveFailures∼1+Protocol+(1|SubjectID) versus ConsecutiveFailures∼1+(1|SubjectID): p < 1e−10, effect of barrier in number of failed attempts after last reward = 0.59 ± 0.02, n = 20 subjects, likelihood ratio test on ConsecutiveFailures∼1+Protocol+Barrier+(1|SubjectID) versus ConsecutiveFailures∼1+Protocol+(1|SubjectID): p < 1e−10).

### OFC, but Not ACC, Is Necessary for the Correct Inference Process

**Figure 5:**
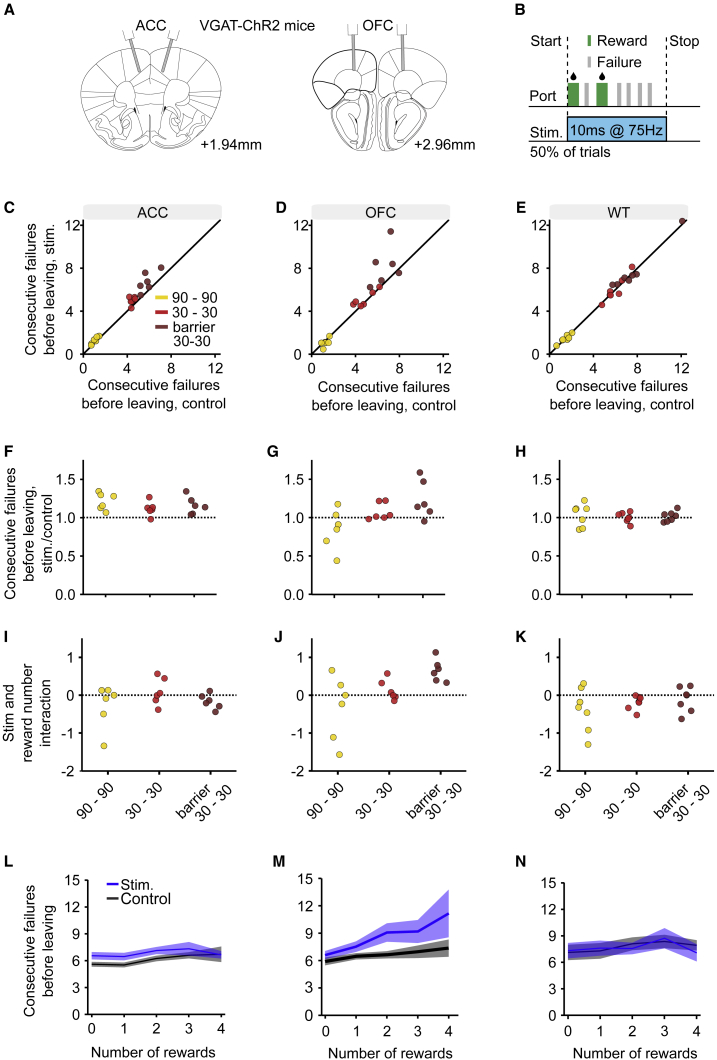
OFC, but Not ACC, Is Necessary for Optimal Inference (A) Scheme of the optic fiber placement. (B) Bilateral photostimulation at 3 mW happened during nose-poking: it was triggered by the first poke in 50% of trials and lasted for 500 ms after the last poke in the trial. (C–E) Consecutive failures before leaving split by environment statistics, barrier condition, and subject inactivation versus control trials, for ACC implanted heterozygotes (C), OFC implanted heterozygotes (D), and wild-types (E), respectively. (F–H) Ratio of consecutive failures before leaving split in the same way as (C)–(E) for ACC implanted heterozygotes (F), OFC implanted heterozygotes (G), and wild-types (H), respectively. When predicting renormalized consecutive failures, *Stimulation* and *Protocol* interact for OFC implanted heterozygotes (p < 1e−10, n = 6 mice) but not for ACC implanted heterozygotes (p = 0.15, n = 6 mice) or wild-types (p = 0.77, n = 7 mice). (I–K) An animal by animal quantification: the coefficient of the interaction term in ConsecutiveFailures∼1+Stimulation+Rewardnumber+Rewardnumber&Stimulation for ACC implanted heterozygotes (I), OFC implanted heterozygotes (J), and wild-types (K). (L–N) Number of consecutive failures as a function of reward number in the 30-30 barrier protocol for ACC implanted heterozygotes (L), OFC implanted heterozygotes (M), and wild-types (N). Solid lines and shaded areas represent mean and SEM across animals. See also Figure S2 and Table S1.

Finally, to study the brain mechanisms of inference in this task, we tested the involvement of different regions of prefrontal cortex by silencing them using optogenetic stimulation of inhibitory GABAergic interneurons in VGAT-ChR2 mice (mice expressing the excitatory opsin channelrhodopsin-2 in inhibitory GABAergic neurons). We examined 19 mice. Nine were bilaterally implanted with optic fibers (Table S1) in the ACC (Figures 5A and S2A), six of these mice were ChR2-expressing (HET) and three were control wild-type littermates (WT) implanted and stimulated in the same manner. Ten (six HET and four WT) were bilaterally implanted in the OFC (Figures 5A and S2B). Transient inactivation of ACC (3 mW power per fiber, 10 ms pulses at 75 Hz, during poking; triggered by the first poke in 50% of trials and maintained for 500 ms after each poke in the trial; Figure 5B) significantly increased the average number of consecutive failures before leaving (Figure 5C, effect of stimulation on consecutive failures after last reward = 0.48 ± 0.05, n = 6 mice, likelihood ratio test on ConsecutiveFailures∼1+Protocol+Stimulation+(1|MouseID) versus ConsecutiveFailures∼1+Protocol+(1|MouseID): p < 1e−10). The same protocol applied to control mice had no effect (Figure 5E, effect of stimulation = 0.003 ± 0.07, n = 7 mice, likelihood ratio test: p = 0.96). More specifically, we found that ACC inactivation multiplicatively increased the number of consecutive failures before leaving, consistently across protocols and animals (Figure 5F, *protocol* and *Stimulation* interact when predicting *ConsecutiveFailures*, likelihood ratio test: p = 1.46e−6, but not when predicting renormalized *ConsecutiveFailures*, likelihood ratio test: p = 0.15, n = 6 mice).

Transient inactivation of OFC also increased the average number of consecutive failures before leaving (Figure 5D; effect of stimulation on consecutive failures after last reward = 0.41 ± 0.08, n = 6 mice, likelihood ratio test on ConsecutiveFailures∼1+Protocol+Stimulation+(1|MouseID) versus ConsecutiveFailures∼1+Protocol+(1|MouseID): p = 5.38e−7). However, unlike the case for ACC inactivation, it did so in a manner that was dependent on the statistics of the environment. That is, the direction of effect for OFC inactivation actually reversed between easy and difficult protocols (effect of stimulation in hard protocol = 1.52 ± 0.24, effect of stimulation in easy protocol = −0.19 ± 0.05, n = 6 mice). This suggests that these two brain areas are differentially involved in the task.

To further investigate the involvement of these prefrontal areas in the inference process and task space representation, we considered a key difference in prediction between the inference model and the simpler stimulus-bound model: the effect of rewards on behavior. As noted above, under normal conditions, rewards fully reset the accumulation process, so that leaving times are not affected by the number of previous rewards (Figure 1E). Strikingly, we found that OFC, but not ACC inactivation, disrupted this pattern: in OFC-inactivated trials, animals became sensitive to the number of rewards: the more rewards gained, the more delayed leaving decisions became (Figures 5I and 5J; for ACC, interaction effect of stimulation and reward number = −0.038 ± 0.07, n = 6 mice, likelihood ratio test on

$$\mathrm{ConsecutiveFailures}∼1+\mathrm{Protocol}+\mathrm{Stimulation}+\mathrm{Stimulation}\&\mathrm{Protocol}+\mathrm{RewardNumber}+\mathrm{Stimulation}\&\mathrm{RewardNumber}+\left(1|MouseID\right)$$

versus

$$\mathrm{ConsecutiveFailures}∼1+\mathrm{Protocol}+\mathrm{Stimulation}+\mathrm{Stimulation}\&\mathrm{Protocol}+\mathrm{RewardNumber}+\left(1|MouseID\right)$$

p = 0.58; for OFC, interaction effect of stimulation and reward number = 0.36 ± 0.1, n = 6 mice, likelihood ratio test: p = 0.0003; triple interaction term of stimulation, reward number, and fiber location = 0.47 ± 0.11, likelihood ratio test: p = 3.44e−5). This pattern of behavior (illustrated in Figures 5L and 5M) is similar to the one observed in naive mice first introduced to this task (Figures 2D and 2E) and is indicative of a less effective stimulus-bound strategy. Thus, the OFC is crucial for behavioral strategies in foraging environments in which states are hidden and require inference based on noisy observations.

## Discussion

In this study, we developed a task in which subjects had to alternate between two foraging sites, only one of which was active at any given moment. The task embodied an important form of non-sensory uncertainty because the active port only delivered rewards with a certain probability. The task thus required subjects to infer whether each omitted reward was simply a stochastic failure or was instead an actual switch of state, offering us a way to directly test whether they have the ability to perform state inference. To solve this task optimally, subjects were essentially required to infer a hidden state of the world (i.e., which site is active) rather than directly assigning a value to each foraging site, as would be optimal, for example, in a matching task (Herrnstein, 1961, Sugrue et al., 2004). We found that both mice and humans displayed hallmarks of optimal, inference-based behavior, reaching very similar solutions.

Our analysis of the behavioral data, particularly the number of consecutive non-rewarded tries before leaving, revealed that leaving decisions agreed with normative predictions of an inference-based foraging strategy in four important ways: (1) the number of consecutive failures was positively correlated with the propensity to leave; (2) rewards had a resetting effect on the leaving decision process; (3) subjects were sensitive to quantitative changes in the statistics of the foraging site; and (4) subjects were sensitive to the travel cost. However, mice and humans differed in an important way: while it took around 6 days for rodents to understand the environment statistics and integrate trial history correctly, humans started displaying hallmarks of the optimal behavior already during the first session. This difference may be due to faster learning but could also reflect the ability to generalize prior structural knowledge relevant to the task.

The accumulation of evidence is considered a primary cognitive computation. Similarly to sensory-guided tasks, in which integration of sensory evidence over time is needed to “average out” stimulus noise (Brunton et al., 2013, Gold and Shadlen, 2007, Shadlen and Newsome, 2001), here too, repeated sampling is needed to determine which of two sites is currently rewarding. Specifically, each failure conveys ambiguous information, as it may be due to either an unlucky attempt at the rewarding site, or a guaranteed failure in the non-active site, and it is only by counting (integrating) the number of consecutive failures that a more accurate state estimation can be made. Our analysis of the leaving probability revealed that, much like in sensory-based tasks, subjects do integrate this information when deciding whether to stay or leave. Moreover, by changing reward and transition probabilities, we were able to precisely control the amount of information associated with each failure, and observed that subjects readily adapted their leaving decisions to these changing conditions, such that the lower the information content of each sample was, the more such samples were needed before leaving.

In the framework of reinforcement learning under uncertainty, given the entire task history—i.e., the sequence of rewards and failures at each port since the beginning of the task—the optimal agent needs to compute a low-dimensional state representation (Niv, 2019) that is most informative of future events. For a formal definition, see Sect. 17.3 in Sutton and Barto (2018). We considered two distinct algorithms for doing this. In the “inference-based” algorithm, we hypothesize that animals recover a meaningful state representation that allows them to take most advantage of the task structure. The current state is represented by the posterior probability of being at the active site given the task history, which in practice is a function of the number of consecutive failures since the last reward. In this algorithm, the final learned solution is optimal and independent of the learning rate used during training. Alternatively, in the “stimulus-bound” algorithm, the animal only uses observable states based on currently available perceptual information (Wilson et al., 2014). The entire task history is summarized by the current location of the animal (left or right site). In the hidden state task, this representation only allows for suboptimal stable solutions, i.e., policies that depend on the site, but not on the reward history.

The primary distinction between sub-optimal, stimulus-bound and optimal, inference-based strategies lies in the impact consecutive rewards have on leaving decisions. In a stimulus-bound behavior, which assigns values directly to the foraging site, the more consecutive rewards are gained at a given site, the higher the value of staying becomes, and consequently, leaving decisions tend to be delayed. In contrast, optimal inference in this task requires ignoring the number of consecutive rewards, because the delivery of a single reward is sufficient to know for certain which site is currently rewarding. As shown in Figure 2, we found that, although initial behavior appeared to be sensory bound, after learning, subjects’ leaving decisions became independent of the number of rewards, consistent with an inference-based approach to leaving decisions. We presume that the change in behavior (between stimulus-bound and inference-based decisions) over the course of training reflects learning, but not that the change in performance necessarily reflects a change in learning rate. What we posit is that two different behavioral controllers, one stimulus-bound and one inference-based, exist, which might correspond to a striatal system and a prefrontal system respectively. Over the course of training, the inference-based controller learns the structure of the task—the correct state representation—through a slow process. In parallel, the inference-based controller’s contribution to behavioral choices is increased over training. This scheme is similar to what was proposed by Daw et al. (2005). Alternatively, the change in behavior over time could be accomplished by meta-learning of hyper-parameters: a more complex stimulus-bound agent could keep track of two different learning rates, one for reward and one for failures. With training, the agent would learn that the optimal reward learning rate is one (complete reset) whereas the failure learning rate is adjusted over time to account for different protocols. Even though this algorithm is distinct, it still requires the ability to adjust a failure learning rate in such a way that it is big for informative failures (in easy protocols) and small for uninformative ones (in harder protocols). Consequently, it results in a computation analogous to the inference model.

Recent accounts (Niv, 2019, Schuck et al., 2016, Stalnaker et al., 2015, Wilson et al., 2014) proposed that the OFC is crucial for accurate state representations, particularly when states are hidden (that is, not explicitly given by the presence of a sensory cue, for example) and have to be inferred from fuzzy evidence. Our findings mesh well with this theory, because we found that OFC, but not ACC, inhibition disrupted inference-based behavior. Unlike under control conditions, in which the number of failures before leaving was independent of the number of previously gained rewards, inhibiting the OFC resulted in mice performing more failures when experiencing large amounts of reward. This latter pattern is consistent with a stimulus-bound strategy, and suggests the possibility that the stimulus-bound strategy serves as a default behavioral approach, and is suppressed by the OFC when inference-based behavior is required. The observation that naive mice behave very similarly to OFC-inactivated mice supports this idea. Several specific computational roles of OFC could account for this effect. OFC could be encoding the representation directly or be necessary to access or update such representation. Alternatively, the representation could be still available, even when the OFC is inactivated, but the region would be responsible for computing the posterior of the hidden states given the representation.

The ACC has been implicated, in Pavlovian and operant tasks, as a potential candidate for the implementation of integration-to-threshold models. In Kawai et al. (2015), the authors observe neurons in the primate ACC whose firing rate scales with the number of consecutive negative outcomes in a Pavlovian task. In the setting of an operant task, Sarafyazd and Jazayeri (2019) showed that such negative outcome accumulation is modulated by the error type (the more surprising the error, the stronger the response) and that microstimulation of ACC accelerates the detection of a context switch. From the foraging perspective, Hayden et al. (2011) reported cells in the ACC encoding the value of a depleting option. However, ACC inactivation in our task, unlike OFC inactivation, had only a modulatory effect on behavior: we did not observe qualitative changes in the strategy of the animals, but only an overall tendency to stay longer at the current port, which interacted multiplicatively both with the task statistics and with increased travel times. The potential activation of neurons in regions immediately adjacent to ACC (e.g., prelimbic cortex or secondary motor cortex) is possible (Figure S2C). However, the areas targeted in the two experiments (ACC and OFC) are considerably further apart (Figure S2C). Because we observed a double-dissociation of effects it is unlikely that the fields of neurons activated across these two experiments were substantially overlapping. Although caution is required in comparing primate and rodent ACC, given the reported anatomical (Uylings and van Eden, 1990) and functional differences (Narayanan et al., 2013, Seamans et al., 2008), our results seem most compatible with the idea that the ACC encodes the value of alternative options, as proposed in Kolling et al. (2016), while not having a primary role in the computations required for the state inference process.

By developing a human video game and a rodent task requiring the same underlying computation to be solved, we could compare computational and cognitive processes across species. From a theoretical standpoint, this strengthens the generality of those results that held true for the two species, such as the ability to infer the hidden structure of the environment and to tune behavior to environmental statistics. From a practical standpoint, the hidden state foraging task makes it possible to use rodent experimentation to more closely guide human clinical research into the mechanisms of manipulations (e.g., drugs) or conditions (e.g., depression) that may affect processes such as state inference.

## STAR★Methods

### Key Resources Table

**Table:** 

REAGENT or RESOURCE	SOURCE	IDENTIFIER
**Chemicals, Peptides, and Recombinant Proteins**		

DAPI	SIGMA ALDRICH	Cat#D9542; RRID:AB_2801570

**Experimental Models: Organisms/Strains**		

C57BL/6NCrl	Charles River Laboratories	strain code: 027; RRID:IMSR_CRL:475
Dat-Cre	Jackson Laboratory	stock number: 006660; RRID:IMSR_JAX:006660
Gad-Cre	Jackson Laboratory	stock number: 010802; RRID:IMSR_JAX:010802
VGAT-ChR2	Jackson Laboratory	stock number: 014548; RRID:IMSR_JAX:014548
FI12-Cre	Mutant Mouse Regional Resource Centers	stock number: 017262-UCD; RRID:MMRRC_017262-UCD
Sert-Cre mouse line 61	Mutant Mouse Regional Resource Centers	stock number: 017260-UCD; RRID:MMRRC_017260-UCD

**Software and Algorithms**		

ImageJ	Schneider et al., 2012	https://imagej.nih.gov/ij/
Construct 2	Scirra Ltd.	https://www.scirra.com/construct2
Julia language	Bezanson et al., 2017	https://julialang.org/
MixedModels.jl	Bates et al., 2019	https://github.com/JuliaStats/MixedModels.jl

**Other**		

Arduino Mega 2560 r3	Arduino	A000067
Pokes detector and valve controller	Champalimaud Hardware Platform	Mice poke simple v1.1
Arduino ports interface	Champalimaud Hardware Platform	Arduino baseboard v2.2

### Lead Contact and Materials Availability

Further information and requests for reagents may be directed to, and will be fulfilled by the Lead Contact, Zachary Mainen (zmainen@neuro.fchampalimaud.org). This study did not generate new unique reagents.

### Experimental Model and Subject Details

#### Mice

Fifty-seven adult male C57BL/6 mice were used in this study. For the inference-based versus stimulus-bound behavior experiment (Figure 2) 18 C57BL/6NCrl wild-type mice of two months age were used. For the protocols manipulation experiment (Figure 3), 20 wild-type animal from different genetic backgrounds (8 Dat-Cre ; 5 Gad2-Cre; 5 Sert-Cre; 1 VGAT-ChR2; 1 F512-Cre) of 6-8 months age were used, in order to reduce animal usage. For inactivation of anterior cingulate or orbitofrontal cortices (Figure 5), 12 VGAT-ChR2 and 7 wild-type littermates were used. Mice genotypes were determined based on PCR and further verified using histological inspection of YFP expression which led to the exclusion of a single ACC implanted animal from further analysis (see Figure S2d, e). The C57BL/6NCrl line was obtained from the Charles river laboratories, breeders were ordered and bred in-house for a maximum of 4 generations or 2 years (strain code: 027). The Dat-Cre mouse line was obtained from the Jackson laboratory (stock number: 006660). The Gad2-Cre was obtained from the Jackson laboratory (stock number: 010802). The Sert-Cre mouse line 61 was obtained from the Mutant Mouse Regional Resource Centers (stock number: 017260-UCD). The VGAT-ChR2 mouse line 8 was obtained from the Jackson laboratory (stock number: 014548). The FI12-Cre mouse line was obtained from the Mutant Mouse Regional Resource Centers (stock number: 017262-UCD). All experimental procedures were approved and performed in accordance with the Champalimaud Centre for the Unknown Ethics Committee guidelines and by the Portuguese Veterinary General Board(Direcao-Geral de Veterinaria, approval 0421/000/000/2016). The mice were kept under a normal 12 h light/dark cycle, and training, as well as testing, occurred during the light period. Before testing or after surgeries, for the inactivation experiments, mice were single-housed. During training and testing the mice were water deprived, and water was available to them only during task performance. Food was freely accessible to the mice in their home cages. Extra water was provided if needed to ensure that mice maintain no less than 80% of their original weight. For the protocols manipulation experiment behavioral training lasted 12 sessions, once per day, followed by 2 days of rest at the end of which we commenced testing. During training mice were exposed to the 3 different protocols (Easy: $p_{RWD}=0.9$ and $p_{SW}=0.9;$ Medium: $p_{RWD}=0.9$ and $p_{SW}=0.3;$ Hard: $p_{RWD}=0.3$ and $p_{SW}=0.3$) for 4 consecutive days (1 day of adaptation and 3 of testing) before transitioning to the next environment. During testing, mice performed 1 session per day, 6 or 7 days a week. In protocols manipulation experiments, the sequence of protocols was counterbalanced across 2 groups of 10 mice (Group A: Hard, Medium, Easy; GroupB: Easy, Medium, Hard). In our analyses we considered 50 poke bouts per session after the first 10 during testing days and excluded poke bouts with no rewards.

#### Human participants

20 right handed healthy adults of Portuguese nationality (10 female and 10 male; 22 to 31 years of age), with no history of psychiatric diagnosis or prescribed drugs in the last 6 months, participated in this study. All participants gave written informed consent, and the study was conducted in accordance with the guidelines of the local ethics committee. The task consisted of 2 sessions of 1 hour, performed in different days with 2 to 10 days in between sessions. Each session consisted of 4 blocks with different protocols, and 10 minutes break after the second block. The sequence of protocols consisted of a block (Medium environment) followed by a short break (2 minutes), then a second block (Easy or Hard environment) followed by a long break (10 minutes), then a third block (Medium environment) followed by a short break (2 minutes) and a final block (Hard or Easy environment). The sequence of environments during testing was counterbalanced across 2 groups as described in mice experiments. In our analyses we considered all tapping bouts after the first 10 and excluded bouts with no rewards.

### Method Details

#### Mice behavioral apparatus

The behavioral apparatus for the task was adapted from the design developed by Zachary F. Mainen and Matt Recchia (Island motion corporation, Nesconset, NY), originally developed for rat behavior. The behavioral box (15 × 12 × 18 cm, model 003102.0001, Island motion corporation), contained 3 front walls (135-degree angle between the center and the side walls) with 2 nose-poke ports attached to the left and right front walls. For the inference-based versus stimulus-bound behavior experiment (Figure 2), we used a custom-made acrylic replicate of the box (15 × 16 × 20 cm). Each port was equipped with infrared emitter/sensor pairs to report the times of port entry and exit (model 007120.0002, Island motion corporation). A nose-poke was considered valid if the infrared beam was broken for at least 100 ms. Water valves (LHDA1233115H, The Lee Company, Westbrook, CT) were calibrated to deliver a drop of 6 μl water for rewarded pokes in Easy and Hard environments and 2 μl of water in Medium environment: the reward size was adjusted to keep the reward amount per correct trial constant. The average number of rewarded attempts per correct trial is $p_{RWD}/p_{SW}$, that is to say 1 in the easy and hard protocol (reward magnitude = 6 μl, amount of water per correct trial = 6 μl) and 3 in the medium protocol (reward magnitude = 2 μl, amount of water per correct trial = 6 μl). In optogenetic experiments, all protocols had an average of one reward per trial, but the reward size was kept at 4 μl to increase the trial number. In optogenetic experiments, blue LEDs were placed in the box ceiling and in all the ports to deliver a masking light. All signals from sensors were processed by Arduino Mega 2560 microcontroller board (Arduino, Somerville, US) and output from the Arduino Mega 2560 microcontroller board was implemented to control water and light delivery. Arduino Mega 2560 microcontroller was connected to the sensors and controllers through an Arduino Mega 2560 adaptor board developed by the Champalimaud Foundation Scientific Hardware Platform. An example behavioral video is available in the supplemental information.

#### Human video game task

Human subjects played a video game on a touchscreen device, with analogous features to the rodent behavioral assay. In the game, subjects receive verbal instructions on how to control a character - a “witch” - on its quest to find and defeat an enemy that hides behind a castle. The witch must walk along the wall of a castle, shooting either the left or the right edge of this wall in search of the enemy that hides, at any given moment, in one of these two edges. The game obeys the same statistics as the rodent task: hitting the enemy is analogous to a water reward, the current location of the enemy corresponds to the active site, and every shot at the active site hits the enemy with probability $p_{RWD}$. Moving between the two sides of the wall has an associated cost (travel cost) that can also be manipulated with the appearance of rougher terrain (analogous to the physical barrier) that diminishes the traveling speed. As in the mouse case, reward size was manipulated to keep the average reward per correct bout constant (3 points). The game ended either when subjects collected 280 points or when a time limit of 20 minutes was exceeded.

Different environments had minor changes in the background images between them - for the medium protocol since it was experienced twice per session, two different backgrounds were used. After the player transitioned to a different site, the enemy was displayed to cue whether the transition had been correct or if instead, the player had to return to the previous site.

The human task was made using custom software developed using the game engine Construct2 (Scirra Ltd., Studio 117, The Light Bulb 1 Filament Walk Wandsworth, London, UK). Graphics were made by Shira Lottem and Tiago Quendera using Inkscape: Open Source Scalable Vector Graphics Editor. Audio assets were made by Tiago Quendera using Audacity(R) except for the Wilhelm Scream (Wikimedia Commons).

An example video (not from an experimental subject) showing the different environments is provided in the supplemental material. The task, open-source code and all assets are available at https://github.com/quendera/human-foraging.

#### Optogenetic stimulation

In order to optically stimulate ChR2 expressing VGAT-expressing GABAergic interneurons we used blue light from a 473 nm laser (LRS-0473-PFF-00800-03, Laserglow Technologies, Toronto, CA or DHOM-M-473-200, UltraLasers, Inc., Newmarket, CA) that was controlled by an acousto-optical modulator (AOM; MTS110-A1-VIS or MTS110-A3- VIS, AA optoelectronic, Orsay, FR) to deliver light 10 ms pulses of light at 75 Hz, connected to Arduino Mega 2560 microcontroller board (Arduino, Somerville, US). Light exiting the AOM was focused into an optical fiber patch cord (200 μm, 0.22 NA, Doric lenses Inc, 357 rue Franquet, Quebec, Quebec, CA), connected to a second fiber patch cord through a rotary joint (FRJ 1x1, Doric lenses), which was then connected to the chronically implanted optic fiber cannula (MFC_200/230-0.48_3mm_ZF1.25(G)_FLT; Doric lenses Inc, 357 rue Franquet, Quebec, Quebec, CA). We estimated an average 15% loss of light power between the patch cord tip and the optic fiber cannula before surgery. In order to deliver light at 3 mW power, previously to each experiment day, the laser power at the tip of the patch cord was adjusted to 3.6 mW, to account for the estimated power loss. To test each protocol, we habituated animals to the new protocol for two days, then stimulated during six consecutive days. Stimulation was delivered on 50% of trials and started with the first valid nose-poke (that is to say after the infrared beam was broken for at least 100 ms). Stimulation ended if the animal did not nose-poke for 500 ms but would restart in case of another valid nose-poke on the same site.

#### Surgical procedures

Animals were anesthetized with isoflurane (4% induction and 0.5 - 1% for maintenance) and placed in a motorized computer-controlled Stoelting stereotaxic instrument with mouse brain atlas integration and real-time visualization of the surgery probe in the atlas space (Neurostar, Sindelfingen, Germany;https://www.neurostar.de). Antibiotic (Enrofloxacin, 2.5-5 mg/Kg, S.C.) and pain killer (Buprenorphine,0.1 mg/Kg, S.C.) and local anesthesia over the scalp (0.2 ml, 2% Lidocaine, S.C.) were administered before incising the scalp. Target coordinates were 1.9 mm A.P., ± 0.5 mm M.L., 1.75 mm D.V. for ACC and 2.9 mm A.P., ± 1.25 mm M.L., 1.8 mm D.V. for OFC. Two craniotomies were performed above the target’s coordinates for OFC implants. For ACC implants fiber were implanted over the target with an angle of ± 16◦ on the ML axis to avoid damage to the superior sagittal sinus, and two craniotomies were performed at coordinates 1.9 mm A.P., ± 1 mm M.L. An optical fiber (200 μmcore diameter, 0.48 NA, 510 mm) housed inside a connectorized implant (M3, Doric lenses, Quebec, Canada) was lowered into the brain (0 degree angle for OFC and 11 degree angle for ACC), through the craniotomy as the viral injection, and positioned10 μmabove the target. The implant was cemented to the skull using Super Bond C&B (Morita, Kyoto, Japan) and once dried covered with black dental cement acrylic (Pi-Ku-Plast HP 36, Bredent, Senden, Germany). The skin was stitched at the front and rear of the implant. Gentamicin(48760, Sigma-Aldrich, St. Louis, MO) was topically applied around the implant. Mice were monitored until recovery from the surgery and returned to their home cage where they were housed individually. Gentamicin (48760, Sigma-Aldrich, St. Louis, MO) was topically applied around the implant. Behavioral testing started at least 1 week after surgery to allow for recovery.

#### Histology and microscopy

For histological analysis mice were perfused transcardially with 4% paraformaldehyde (PFA) in phosphate buffer solution (PBS). After removing the brain they were left for 24 hours in 4% PFA solution in PBS, then transferred in 0.1% sodium azide solution in PBS. Brains were sliced in 50 um coronal sections on a vibratome (Leica VT 1000 S), collected in wells maintaining the anterior-posterior order, and finally mounted on microscope slides (Thermo scientific, superfrost plus), with mowiol.

Fluorescent images were acquired with an automated slide scanner (AxioScan Z1) equipped with a 10x, 0.45 NA PlanApochromat objective and a Hamamatsu OrcaFlash camera. Use of the appropriate filter combination allowed for DAPI and EYFP acquisition (Beam Splitter: 395, excitation: 330-375, emission: 430-470, and Beam splitter: 498, excitation: 453-485, emission: 507-546 respectively).

Optic cannula placement was determined using coronal sections of the prefrontal cortex through which the fiber tract was visible. We determined the position by locating the section with the broadest base of the cannula tract and comparing the DAPI staining with the Allen Mouse Brain Atlas (Lein et al., 2007) (Figure S2; Table S1).

### Quantification and Statistical Analysis

#### Statistical analysis

The statistical analysis, which can be found in the Results section and figure legends, was performed using mixed effect models (McLean et al., 1991), in particular the Julia (Bezanson et al., 2017) implementation MixedModels.jl (Bates et al., 2019). For each mixed model, we report the maximum likelihood estimate of the coefficient of interest ± the standard error of the estimate. Our N is the number of subjects: as different experiments had a potentially different number of subjects, we report it after every statistical test. We fitted models with a random intercept (depending on subject identity) and compared nested models using a likelihood ratio test: in particular we used a chi-square test on the difference of the deviance of the two nested models, using as many degrees of freedom as the difference between the number of degrees of freedom of the two nested models (Wilks, 1938). That is to say, given two models m and n where n is a special case of m:

$$p=1-cdf\left(\chi_{dof\;\left(m\right)-dof\;\left(n\right)}^{2},deviance\left(n\right)-deviance\left(m\right)\right)$$

When the p value is too small, we do not report the value but simply write p < 1e-10, which is floating point notation for p < 10^−10^. To describe mixed models we will use Wilkinson notation (Wilkinson and Rogers, 1973), with | denoting random effects and & denoting interaction terms. For example the formula:

ConsecutiveFailures∼1+Protocol+Stimulation+(1|MouseID)

uses as predictor for the number of consecutive failures after last reward a constant intercept, a coefficient for each protocol different than the medium protocol (which we consider as baseline), a coefficient for stimulation and a random intercept across mice. The formula

ConsecutiveFailures∼1+Protocol+Stimulation+Protocol&Stimulation+(1|MouseID)

would also allow for an interaction term between protocol and stimulation.

We did not test whether the data met the assumptions of the statistical methods used.

#### Task design

We designed a probabilistic foraging task for parallel use in mice and humans. Subjects sought rewards (water or points, respectively), by actively probing a foraging site (nose-poking or screen-tapping, respectively). Each site could be in one of two states, active or inactive. Each try in the active state yielded reward with probability $p_{RWD}$, and could cause the site to switch to the inactive state with probability $p_{SW}$. This required the subjects to travel to a second, fresh, site at some distance and bear a travel cost. Subjects were therefore tasked with inferring a hidden state (active or inactive) through a stochastic sequence of observations (rewards and failures).

#### Relevant statistics in the task

After a rewarded attempt, the subject could be sure to be in the correct location: ambiguity comes from failures, as it was possible that the target was correct but the subject was being unlucky. The more unsuccessful attempts, the higher the probability of a transition having occurred. Accumulated evidence in favor of a switch is a monotonically increasing function of the task parameters $p_{RWD}$ and $p_{SW}:$ the higher the reward probability, the more informative a failure is. Trivially, the higher the switch probability, the more likely the switch.

#### Possible task space representations

When analyzing our task, we consider two possible state representations. One is simpler and analogous to traditional approaches to modeling n-armed bandit tasks: the state corresponds to the current location of the subject (i.e., one of the two reward sites). The value of the two sites changes over time, yet the animals may be able to track this change with fast model-free learning. A second, more principled but more abstract approach, postulates that the subjects tries to infer the optimal state representation, i.e., the probability that their current location is rewarding. In this model, there is no longer any need for fast online learning as the task representation is stable. The computation happening in real time is the inference process to compute this probability. To account for variability in the behavior, we allow both decision noise, distributed according to the soft-max rule, as well as inference noise (the inference process may be suboptimal). We will refer to these two learning paradigms as stimulus-bound learning and inference-based learning respectively. It is important to note that, given the richer state representation, inference-based learning is the optimal way to solve the task and clearly outperforms simpler heuristics such as stimulus-bound learning.

#### Stimulus-bound learning

In stimulus-bound learning, we first define the relative value V as the difference of the value of the leftport and the value of the right port:

(S1) $$V=V_{LEFT}-V_{RIGHT}$$

We defined two auxiliary variables: a reward variable r indicating the outcome of each reward attempts, i.e., 1 for a reward and 0 for a failure, and a site variable s, indicating the current site, 1 for left and −1 for right.

We can define a signed outcome o of each reward attempt, which is:

(S2) o=r·s

that is to say, 1 for a reward on the left, −1 for a reward on the right and 0 for an omission.

For any attempt, we can then update the relative value using the signed outcome and some discount parameter γ

(S3) $$V_{t+1}=\left(1-\gamma \right)V_{t}+\gamma o_{t+1}$$

Which admits an explicit solution:

(S4) $$V_{t}={\left(1-\gamma \right)}^{t}\cdot V_{0}+\gamma \cdot \sum_{i=1}^{t}{\left(1-\gamma \right)}^{t-i}\cdot o_{i}$$

The probability of staying is a monotonically increasing function of the value of staying, so that rewards should make the animal more likely to stay and omissions more likely to leave, in a symmetric way.

#### Inference-based learning

We first derive recursive formulas to compute the probability that the current site is not rewarding as a function of the sequence of successful and failed reward attempts performed by the subject. In this model, the subject would compute the relative value as a function of the probability of the left (or right) site being active given the task history ($r_{1},\;\;\;\ldots ,\;\;\;r_{t}$ represent the outcomes of the various attempts and $s_{1},\;\;\;\ldots ,\;\;\;s_{t}$ the site of each attempt):

(S5) $$V_{t}=p_{RWD}\left(P\left(Lef\;tActive|r_{1},\;\;\;s_{1},\;\;\;\ldots ,\;\;\;r_{t},\;\;\;s_{t})-P(RightActive|r_{1},\;\;\;s_{1},\;\;\;\ldots ,\;\;\;r_{t},\;\;\;s_{t}\right)\right)$$

#### From value to decision

We have now defined to different ways to compute the relative value of left versus right, one directly based on reward accumulation, and one based on evidence accumulation. To define a behavior from this relative value, we need to consider two more parameters. First of all we need a bias term □: as the two foraging sites are far apart, subjects should prefer to repeat site rather than alternate, to avoid the travel cost. Then we need a “inverse temperature” parameter β to describe how deterministic the animal is (with a very high β the animal would almost always choose the option with greater value, whereas with β=0 the animal would choose randomly). We can then use the soft-max rule to generate behavior:

(S6) P(NextLeft|V,s)=σ(β(V+s·T))

where s represents the current site (1 for left and −1 for right).

In the simulations we will use the same softmax rule to simulate behavior: the difference between stimulus-bound and inference-based learning derives from the different procedures used to compute the relative value.

#### Computing the likelihood ratio

In inference-based learning we defined the relative value as a function of the relative difference:

(S7) $$P\left(Lef\;tActive|\;r_{1},\;\;\;s_{1},\;\;\;\ldots ,\;\;\;r_{t},\;\;\;s_{t})-P(RightActive\;|\;r_{1},\;\;\;s_{1},\;\;\;\ldots ,\;\;\;r_{t},\;\;\;s_{t}\right)$$

This quantity can be computed recursively. To do so we will need an auxiliary variable. We define $R_{t}$ as the probability ratio that the current site is active or inactive given task history:

(S8) $$R_{t}=\frac{P(Inactive|r_{1},\;\;\;s_{1},\;\;\;\ldots ,\;\;\;r_{t},\;\;\;s_{t})}{P(Inactive|r_{1},\;\;\;s_{1},\;\;\;\ldots ,\;\;\;r_{t},\;\;\;s_{t})}$$

From $R_{t}$ we can compute $V_{t}$ as follows:

(S9) $$V_{t}=p_{RWD}\cdot \left(1-\frac{2}{R_{t}^{-s_{t}}+1}\right)$$

Rather than computing $V_{t}$ recursively directly, we notice that $R_{t}$ respects a simple recursive equation (likelihood ratio update equation):

(S10) $$R_{t+1}={\left(\frac{R_{t}+p_{SW}}{1-p_{SW}}\right)}^{s_{t}s_{t+1}}\cdot \frac{P\left(r_{t+1}|Inactive\right)}{P\left(r_{t+1}|Active\right)|}$$

where $p_{SW}$ represents the probability of switching from active to inactive state.

The term $\left(R_{t}+p_{SW}/1-p_{SW}\right)$ is the ratio between the following two equations:

(S11) $$P\left(NextInactive\right)=P\left(Inactive\right)+p_{SW}P\left(Active\right)$$

$$P\left(NextActive\right)=\left(1-p_{SW}\right)\cdot P\left(Active\right)$$

The exponent $s_{t}s_{t+1}$ simply means that the probability ratio (P(Inactive)/P(Active)) inverts when the subject changes site. Finally the term $\left(P(r_{t+1}|Inactive)/P(r_{t+1}|Active)\right)$ represents the new evidence acquired with the outcome of attempt t+1.

In the case of a reward, $P\left(r_{t+1}|Inactive\right)=0$, so:

(S12) $$R_{t+1}=0$$

If $r_{t+1}$ is a failure, then $P\left(r_{t+1}|Inactive\right)=1$ whereas $P\left(r_{t+1}|Active\right)=1-p_{RWD}$, so the likelihood ratio update equation simplifies to:

(S13) $$R_{t+1}={\left(\frac{R_{t}+p_{SW}}{1-p_{SW}}\right)}^{s_{t}s_{t+1}}\cdot \frac{1}{1-p_{RWD}}$$

Having established that $R_{t}$ resets to 0 with a reward, we can analyze the most interesting case for a probability computation: a sequence of attempts on the same site (let us say $s_{1},\;\;\;\ldots \;\;\;s_{t}=1$) where the first attempt is rewarded (thus resetting the probability) and the following are not.

As the first attempt is rewarded, $R_{1}=0$. Furthermore, if we assume that all attempts are on the same site, the likelihood ratio grows following the recursive equation:

(S14) $$R_{t+1}=\frac{R_{t}+p_{SW}}{\left(1-p_{SW}\right)\left(1-p_{RWD}\right)}>R_{t}$$

We can define the auxiliary quantity

(S15) $$\rho =\frac{1}{\left(1-p_{SW}\right)\left(1-p_{RWD}\right)}>1.$$

Our recursive equation becomes:

(S16) $$R_{t+1}=\rho \cdot \left(R_{t}+p_{SW}\right)$$

This is a standard linear recursion that we can solve with linear transformation

(S17) $$S_{t}=R_{t}+\frac{p_{SW}}{\rho -1}$$

The recursion of $S_{t}$ is:

(S18) $$S_{1}=\frac{p_{SW}}{\rho -1}$$

$$S_{t+1}=\rho \cdot S_{t}$$

whose solution is

(S19) $$S_{t}=p_{SW}\frac{\rho^{t-1}}{\rho -1},$$

therefore:

(S20) $$R_{t}=p_{SW}\frac{\rho^{t-1}-1}{\rho -1}$$

That is to say $R_{t}$ grows exponentially with rate $\log\left(\rho \right)=-\log\left(1-p_{RWD}\right)-\log\left(1-p_{SW}\right)$: increasing either $p_{RWD}$ or $p_{SW}$ would increase the growth rate of R.

### Data and Code Availability

All analysis was performed using custom code written in Julia (Bezanson et al., 2017). The code used to simulate value or inference models is available on GitHub, under the MIT license, at https://github.com/piever/ValueInferenceTools.jl. The data is published on Zenodo with DOI 10.5281/zenodo.3607558 and can be found at https://zenodo.org/record/3607558.

## Supplemental Information

Document S1. Figures S1–S2 and Table S1

Document S2. Article plus Supplemental Information
