# Supplementary material for: Inference-Based Decisions in a Hidden State Foraging Task: Differential Contributions of Prefrontal Cortical Areas
Source: Neuron. 2020 Apr 8;106(1):166–176.e6. doi: 10.1016/j.neuron.2020.01.017 (PMC7146546; doi:10.1016/j.neuron.2020.01.017)
Supplement: Document S1. Figures S1–S2 and Table S1 [file mmc1.pdf]

Neuron, Volume *106*

Supplemental Information

Inference-Based Decisions in a Hidden State Foraging Task: Differential Contributions of Prefrontal Cortical Areas

Pietro Vertechi, Eran Lottem, Dario Sarra, Beatriz Godinho, Isaac Treves, Tiago Quendera, Matthijs Nicolai Oude Lohuis, and Zachary F. Mainen

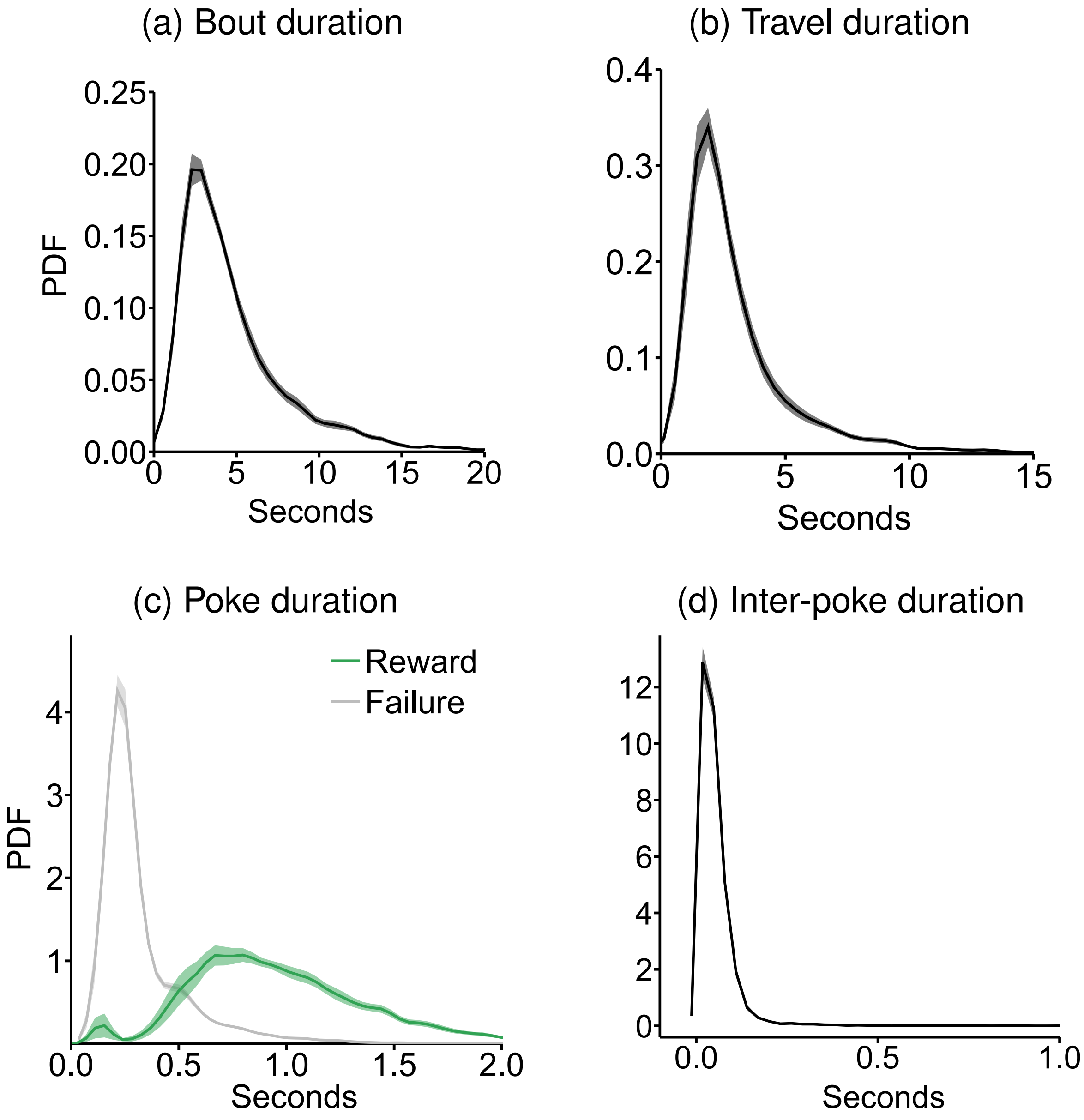

Figure S1. Related to Figure 2. Summary plots of behavioral times for trained animals (day 10 to 12). **a** Duration of poke bouts (from first poke-in to last poke-out before leaving the port). **b** Duration of travel (i.e. from last poke-out to first poke-in of next trial on the other site). **c** Time spent inside the poke for rewarded and unrewarded attempts. **d** Inter-poke interval within pokes on the same site (outliers of duration greater than 3 seconds have been excluded from the analysis for numerical reasons).

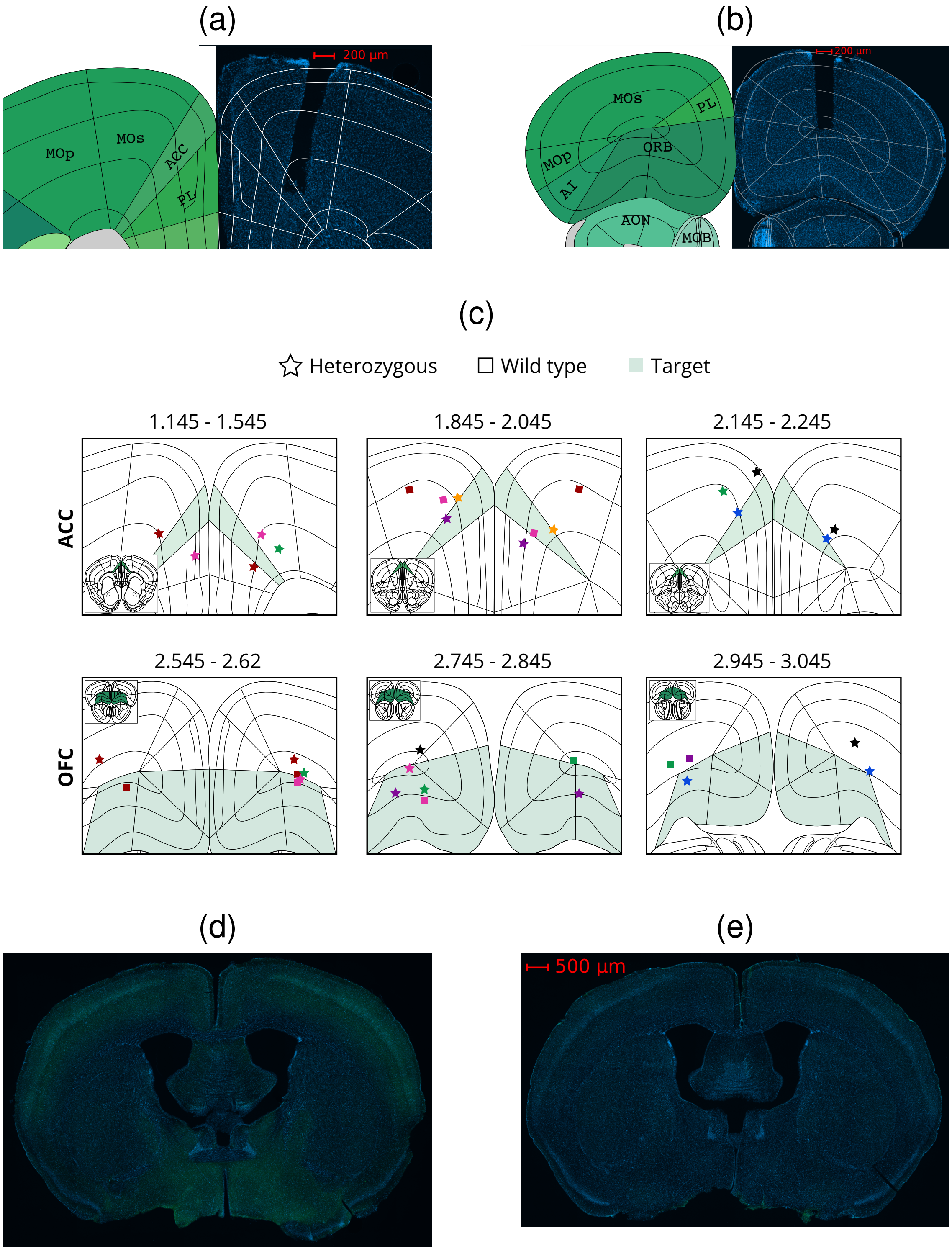

Figure S2. Related to Figure 5. Inhibition of ACC or OFC cortex in VGAT-ChR2 mice. **a, b** Coronal section from 2 VGAT-ChR2 mouse (blue, DAPI). Dark area show the location of the optic cannula over ACC in (**a**) or over OFC in (**b**). Correct positioning of the fiber was verified overlapping reference images from the coronal Allen Mouse Brain Reference Atlas. ACC, anterior cingulate; AI, agranular insular area; AON, anterior olfactory nucleus; CLA, claustrum; DP, dorsal peduncular area; fa, corpus callosum; ILA, infralimbic area; MOp, primary motor area; MOs, secondary motor area; PL, prelimbic cortex; ORB, orbital area; TT, taenia tecta. Image credit: Allen Institute. **c** Fiber tip placement in 9 out of 10 animals (in one wild type animal it was impossible to perform histological controls, see Table S1 for details) implanted in ACC (top row) and in the 10 animals implanted in OFC (bottom row). **d, e** Fluorescence widefield microscopy of EYFP signal conjugated to ChR2 in mouse VGAT-ChR2-EYFP line 8, in an example heterozygote (**d**) and an animal initially genotyped as heterozygous that was excluded from the dataset due to lack of EYFP expression (**e**).

MOUSE ID	SIDE	TARGET	AP	ML	DV	GENOTYPE	EYFP EXPRESSION
B21	Right	N/A	N/A	N/A	N/A	Wild type	N/A
B21	Left	N/A	N/A	N/A	N/A	Wild type	N/A
B22	Right	ACC	2.045	1.17	1.17	Wild type	FALSE
B22	Left	ACC	2.045	-1.17	1.18	Wild type	FALSE
B23	Right	ACC	2.145	0.83	1.99	Heterozygous	TRUE
B23	Left	ACC	2.245	-0.23	1.19	Heterozygous	TRUE
B24	Right	ACC	1.145	0.99	1.88	Heterozygous	TRUE
B24	Left	ACC	2.245	-0.69	1.45	Heterozygous	TRUE
B25	Right	ACC	1.42	0.73	1.66	Heterozygous	TRUE
B25	Left	ACC	1.42	-0.2	1.96	Heterozygous	TRUE
B41	Right	ACC	1.545	0.62	2.12	Heterozygous	FALSE
B41	Left	ACC	1.545	-0.71	1.65	Heterozygous	FALSE
B42	Right	ACC	2.145	0.72	2.12	Heterozygous	TRUE
B42	Left	ACC	2.145	-0.48	1.75	Heterozygous	TRUE
B43	Right	ACC	1.845	0.82	1.75	Heterozygous	TRUE
B43	Left	ACC	1.845	-0.5	1.3	Heterozygous	TRUE
B44	Right	ACC	1.945	0.38	1.93	Heterozygous	TRUE
B44	Left	ACC	1.945	-0.66	1.6	Heterozygous	TRUE
B45	Right	ACC	2.045	0.53	1.79	Wild type	FALSE
B45	Left	ACC	2.045	-0.69	1.32	Wild type	FALSE
P21	Right	OFC	2.62	1.26	2.13	Wild type	FALSE
P21	Left	OFC	2.745	-1.01	2.6	Wild type	FALSE
P22	Right	OFC	2.62	1.35	1.99	Heterozygous	TRUE
P22	Left	OFC	2.745	-1.01	2.44	Heterozygous	TRUE
P23	Right	OFC	2.745	1.22	2.5	Heterozygous	TRUE
P23	Left	OFC	2.745	-1.42	2.49	Heterozygous	TRUE
P24	Right	OFC	2.62	1.26	2.1	Wild type	FALSE
P24	Left	OFC	2.62	-1.19	2.2	Wild type	FALSE
P25	Right	OFC	2.62	1.3	2.08	Heterozygous	TRUE
P25	Left	OFC	2.845	-1.22	2.14	Heterozygous	TRUE
P41	Right	OFC	3.045	1.35	2.03	Heterozygous	TRUE
P41	Left	OFC	3.045	-1.22	2.17	Heterozygous	TRUE
P42	Right	N/A	N/A	N/A	N/A	Wild type	FALSE
P42	Left	OFC	3.045	1.18	1.85	Wild type	FALSE
P43	Right	OFC	2.545	-1.21	1.8	Heterozygous	TRUE
P43	Left	OFC	2.545	1.57	1.8	Heterozygous	TRUE
P44	Right	OFC	2.8455	-1.14	2.04	Wild type	FALSE
P44	Left	OFC	2.945	1.46	1.94	Wild type	FALSE
P45	Right	OFC	3.045	-1.14	1.63	Heterozygous	TRUE
P45	Left	OFC	2.845	1.07	1.88	Heterozygous	TRUE

Table S1. Related to Figure 5 and Figure S2. Optic fibers placement coordinates and expression EYFP. Location of optic fibers across all VGAT-ChR2 animals used for this paper with anterior-posterior (AP), medio-lateral (ML) and dorso-ventral (DV) coordinates, according to the Allen Mouse Brain Atlas (Lein et al., 2007). In mouse B21, it was not possible to perform histological controls, due to the sudden death of the animal after the experiment period, which precluded us from performing the perfusion of its brain. In mouse P42, it was not possible to determine the placement of the fiber in the right hemisphere, due to damage in the slices during cutting. The expression of EYFP conjugated to ChR2 was assessed through widefield fluorescence microscopy to confirm the animal genotype. In mouse B41, EYFP signal was not detected (Fig. S2) despite the fact that the animal was initially genotyped as heterozygous; we therefore excluded it from the analysis.
